# Clinical effect of treating renal transplant recipients with percutaneous coronary intervention and its safety

**DOI:** 10.12669/pjms.322.8952

**Published:** 2016

**Authors:** Yanxuan Zhang, Zhenhong Pan, Jun Fang, Qingshan Qu, Xin Jiang, Ming Li

**Affiliations:** 1 Yanxuan Zhang, MD. Kidney Disease Center, People’s Hospital of Zhengzhou, Henan, 450002, China; 2 Zhenhong Pan, B Med. Kidney Disease Center, People’s Hospital of Zhengzhou, Henan, 450002, China; 3 Jun Fang, MM. Kidney Disease Center, People’s Hospital of Zhengzhou, Henan, 450002, China; 4 Qingshan Qu, B Med. Kidney Disease Center, People’s Hospital of Zhengzhou, Henan, 450002, China; 5 Xin Jiang, MM. Kidney Disease Center, People’s Hospital of Zhengzhou, Henan, 450002, China; 6 Ming Li, MM. Kidney Disease Center, People’s Hospital of Zhengzhou, Henan, 450002, China

**Keywords:** Acute coronary syndrome, Clinical follow up, Major adverse cardiovascular events, Percutaneous coronary intervention, Renal transplant

## Abstract

**Objective:**

To explore clinical effect of treating acute coronary syndrome (ACS) of renal transplant recipients with percutaneous coronary intervention and its safety.

**Methods:**

Forty two renal transplant recipients who were diagnosed with acute coronary syndrome and received percutaneous coronary intervention (PCI) in our hospital were selected. Serum creatinine (Cr) and glomerular filtration rate (GFR) were compared before surgery, 48 ~ 72 hour after surgery and one year after surgery. All patients were followed up.

**Results:**

All patients successfully completed PCI. Contrast-induced nephropathy was not found after surgery. Cr and GFR 48 ~ 72 hour after surgery and one year after surgery had no significant differences with that before surgery (P>0.05). The follow up lasted for (61.2±32.2) months averagely. Of 42 cases, 4 cases died, 6 cases were found with nonfatal myocardial infarction, 4 cases were observed with repeat revascularization and 12 cases had accumulative major adverse cardiovascular events (MACE).

**Conclusion:**

PCI is proved to be effective in treating renal transplant recipients; no severe complications are found and renal function recovers well after treatment.

## INTRODUCTION

Kidney transplant, one of the major treatments aiming at end-stage renal disease, refers to transplant kidney of healthy people to patients who lose renal function due to renal lesions.^1^,^2^ Recently, more and more renal transplant recipients survive after surgery because of the constant development of medical technology and application of novel immunosuppressive drug. However, cardiovascular complication is still the leading cause affecting long-term survival of renal transplant recipients after surgery.^3^,^4^ It has been found that, cardiovascular disease (CVD) is the major cause of renal transplant recipients, and renal transplant recipient usually involves multiple cardiovascular risk factors including traditional risk factors and special risk factors associated to renal failure and transplant.^5^ Coronary artery disease developed by renal transplant recipients is often complex and difficult to be treated. Treatment of CVD especially coronary heart disease has attracted more and more importance.^6^,^7^ Coronary arteriography is the gold standard for diagnosing coronary heart disease. Interventional therapy is an effective way for treating coronary heart disease; however, the safety of interventional therapy for renal transplant recipients has not been studied on a large-scale.

To improve long-term survival rate of renal transplant recipients, this study reviewed 42 renal transplant recipients who received percutaneous coronary intervention (PCI) due to acute coronary syndrome (ACS), compared changes of renal function index before and after PCI and moreover followed up all patients. This work is expected to provide a useful guidance for promotion of PCI in clinic by evaluating clinical effect of PCI in treating renal transplant recipients with acute coronary syndrome (ACS) and its safety.

## METHODS

### Clinical data

Forty two renal transplant recipients who received PCI for treating ACS in cardiovascular center of People’s Hospital of Zhengzhou between March 2011 and June 2014 were selected for this study. It inlcuded 30 males and 12 females, with an average age of 52.4±6.6 years. They received kidney transplant (9.5±3.9) years before. Sixteen cases developed acute myocardial infarction and 26 cases had unstable angina pectoris; 36 cases (86%) had hypertension, 14 cases (33%) had diabetes and 34 cases (81%) had hyperlipemia.

### Method

All patients were treated with hydration therapy. Radial artery or femoral artery on the right side was punctured. Then Judkins method was applied to perform selective coronary angiography, multi-position projection and multi-site angiography. Patient with 50% over coronary artery stenosis was diagnosed having coronary heart disease and patient with 75% stenosis area required stent implantation. Coronary artery stenosis of the patients was recorded. Left main coronary artery stenosis area larger than 50% and left anterior descending branch, left circumflex artery and right coronary artery stenosis area larger than 75% were taken as the evaluation criteria; large branches such as diagonal branch, obtuse marginal branch and right ventricular branch were attributed to left anterior descending branch, left circumflex artery and right coronary artery. Lesions were grouped into triple-vessel lesion, double-vessel lesion and single-vessel lesion. Severity and location of coronary artery lesion as well as condition of interventional treatment were recorded. Besides, coronary angiogram performance of 42 patients was analyzed. Concentration of creatinine (Cr) in serum was measured before PCI, 48 ~ 72 hourh and one year after PCI respectively. Also, glomerular filtration rate (GFR) was calculated by simplified MDRD (Modification of Diet in Renal Disease). All patients were followed up; occurrence of major adverse cardiovascular events (MACE) such as death, repeat revascularization and nonfatal myocardial infarction were observed.

### Statistical method

All data were analyzed with SPSS 19.0 statistics software. Enumeration data were described with number of percentage. Renal function index was compared before and after PCI with paired t test. Difference was considered to have statistical significance if P < 0.05. Curve of survival rate was drafted with Kaplan-Meier method.

## RESULTS

### Coronary angiography and PCI

Coronary angiography confirmed 32 cases of left main artery lesion and (or) three branch lesion (76%), 6 cases of two branch lesion (14%), 4 cases of one branch lesion (10%), 14 cases of artery tortuosity (33%), 12 cases of severe calcification (29%), 20 cases of small vessel lesion (48%), 12 cases of thrombotic lesion (29%) and 2 cases of chronic coronary artery total occlusion (CTO) (5%). Sixty two stents including 54 drug eluting stents and 8 bare metal stents were inserted. Thirty two stents were inserted in left anterior descending branch, 10 in left circumflex artery and 20 in right coronary artery.

### Changes of renal function

Cr and GFR 48 hour ~ 72 hour and one year after PCI were not found to be significantly different compared to before PCI. Moreover, neither of them were observed with contrast-induced nephropathy after PCI. During follow up, patients who required hematodialysis or secondary renal transplantation due to severely deteriorated renal function were also not observed. Table-I shows the comparison of renal function index before and after PCI.

**Table-I:** Comparison of renal function index before and after PCI.

	Cr (μmol/L)	GFR [ml/(min·1.73m^2^)]
Before surgery	122.00 ± 70.12	80.97 ± 34.50
48-72 h after surgery	126.80 ± 67.66	76.82 ± 31.01
One year after surgery	128.13 ± 71.77	78.33 ± 38.00
P1	0.119	0.091
P2	0.142	0.464

P1: comparison of index before PCI and 48 ~ 72 h after surgery;

P2: comparison of index before PCI and one year after PCI. P<0.05 indicates difference was statistically significant.

### Occurrence of MACE

**Fig.1:**
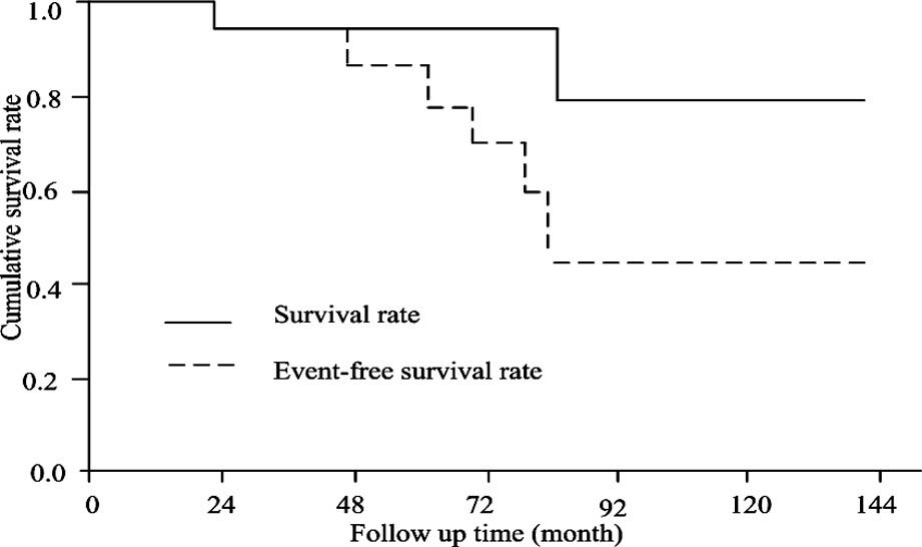
Survival rate and event-free survival rate curve

All 42 patients were followed up after PCI. MACE was not found one year after PCI, but afterwards, it occurred. They were followed up for (61.2±32.2) months on an average after intervention. Of 42 cases, 4 cases died, 6 cases developed nonfatal myocardial infarction, 4 cases were observed with repeat revascularization and 12 cases had accumulative MACE Survival rate curve suggested that, 48-month survival rate was 94.7% and 96-month survival rate was 78.9%; 48-month event-free survival rate was 86.8% and 96-month event-free survival rate was 45.7%. Fig.1.

## DISCUSSION

For renal transplantation recipients, coronary heart disease is the leading risk factor affecting long-term survival.^8^,^9^ About 40% patients are detected with CVD three years after renal transplant. Besides conventional risk factors such as smoking, hypertension, dyslipidemia and diabetes, many other transplant-related risk factors are also involved, leading to higher incidence of coronary heart disease.^10^

Currently, coronary arteriography is not advocated to be performed on renal transplantation recipients in most hospitals. That is because coronary arteriography is not easy to be accepted by most people as it is traumatic and expensive and moreover, function of transplanted kidney may be affected by contrast agent. About 16% to 30% of renal transplant recipients who have not undergone coronary angiography would die of cardiovascular event, and some patients cannot receive timely and effective treatment due to inadequate examination, leading to severe outcome.^11^ A study^12^ has confirmed that renal transplantation recipients with coronary artery stenosis are more likely to die if they receive conventional therapy using drugs compared to those who received revascularization (PCI or coronary artery bypass surgery). Thus kidney disease improving global outcomes (KDIGO) suggested renal transplantation recipients to receive proper therapy to controlling CVD in the clinical guidance for diagnosis and treatment of renal transplantation recipients.^13^ Therefore, patients who have typical clinical symptoms or who are highly suspected with coronary heart disease need to be examined by coronary arteriography and treated with PCI timely.

In this study, differences of Cr and GFR of patients before PCI and 48~72 hour after PCI were not remarkably different and moreover no case was observed with contrast-induced nephropathy, indicating coronary angiography or PCI might have no severe adverse effects on functions of transplanted kidney. But as the sample size was small, its safety remains to be further studied. Chinese experts proposed four principles for preventing contrast-induced nephropathy, i.e., layering, hydration, limited quantity and iso-osmia.^14^ Renal transplantation recipients who are considered to be highly likely to develop contrast-induced nephropathy should be given a proper dosage of contrast agent and adequate hydration, thus to reduce damage of contrast agent on function of transplant kidney. In addition, some drugs such as N-acetylcysteine and vitamin V may be effective in preventing contrast-induced nephropathy;^15^ treatment with statins may plays a protective function on kidney. Even if contrast-induced nephropathy occurs, short-term renal replacement therapy may be useful in recovering renal function.

At present there is no definite diagnosis and treatment guide for renal transplantation recipients who develop ACS. It has been found that, coronary artery bypass surgery or PCI can both benefit patients who develop coronary heart disease in combination with chronic renal disease. Mota et al.^16^ carried out a clinical follow up on 33 renal transplantation recipients who underwent percutaneous coronary and found three patients died due to non-cardiac reason, three patients developed acute coronary syndrome and 9% had in-stent restenosis during follow up (30 months average). Treating renal transplantation recipients with PCI is seldom studied in China. A study carried out by Liu Fengxuan et al.^17^ suggested that 6 renal transplantation recipients were observed with no deterioration on renal function after receiving coronary angiography and PCI with no cardiac events during follow up. The follow up in this study lasted for a longer time. Neither of them developed MACE within one year and 96-month survival rate was 78.9%, indicating PCI was able to improve prognosis of renal transplantation recipients.

## CONCLUSION

PCI is proved to be feasible and safe in treating renal transplantation recipients who had ACS, and it has little influence on renal function. But this study is a single center retrospective review and the sample size is small. Therefore curative effect of treating renal transplantation recipients with PCI and its safety remain to be further explored by carrying out large-scale clinical studies.

## References

[1] Wang XH (2013). International frontier research issue and new progress on renal transplant. Chin J Organ Transplan.

[2] Group of Expert Consensus of Contrast Applying in Intervention of Coronary Artery Disease (2010). Expert consensus of contrast applying in intervention of coronary artery disease. Chin J Cardiovas Res.

[3] Li FX, Zhang MR (2012). Research and prevention of contrast- induced nephropathy during perioperative period. Chin Gener Pract.

[4] Vanrenterghem YF, Claes K, Montagnino G, Fieuws S, Mses B, Villa M (2008). Risk factors for cardiovascular events after successful renal transplantation. J Transplan.

[5] Kahn MR, Fallahi A, Kim MC, Esquitin R, Robbins MJ (2011). Coronary artery disease in a large renal transplant population: implications for management. Am J Transplant.

[6] Liu YF, Liu SR, Liang J, Meng YM, Wu G, Song SW (2003). Treatment of diabetes complicated with diabetic nephropathy by pancreas-kidney transplantation: a report of 13 cases. Chin J Hepatobiliary Surg.

[7] Wang ZJ, Zhou YJ, Liu YY, Zhao YX, Shi DM, Guo YH (2009). Association of chronic kidney disease with clinical outcomes after revascularization for patients with multiple coronary artery disease. Chin J Intervent Cardiol.

[8] Israni AK, Snyder JJ, Skeans MA, Peng Y, Maclean JR, Weinhandl ED (2010). Predicting coronary heartdisease after kidney transplantation: patient outcomes in renal transplantation (PORT) study. Am J Transplant.

[9] Ojo AO (2006). Cardiovascular complications after renal transplantation and their prevention. Transplantion.

[10] Jiang DJ, Hu GP, Fu R, Gao XJ, Jia YL, Wang DD (2007). Intervention therapy on 12 patient with coronary heart disease in combination with serious heart failure. Chin J Cardiol.

[11] Kumar N, Baker CS, Chan K, Duncan N, Malik I, Frankel A (2011). Cardiac survival after pre-emptive coronary angiography in transplant patients and those awaiting transplantation. Clin J Am Soc Nephrol.

[12] Bax JJ, Van der Wall EE (2000). Assessment of myocardial viability: guideto prognosis and clinical management. Eur Heart J.

[13] Zhou B, Deng JH, Zhang W (2007). Ddtection of myocardial ciability using echocardiography and clinical significance. Chin J Cardiovas Med.

[14] Lu MY, Ye HM, Wang WM, Liu J, Zhao H, Ma YL (2012). Clinical efficacy and follow up of drug eluting stent treatment for 120 unprotected left main coronary artery lesions. Chin J Intervent Cardiol.

[15] Jiang L, Shen WF, Zhang JS, Zhang RY, Lv AK, Hu J (2002). Acute and long-term results after coronary stenting in patients with multivessel coronary artery disease. J Clin Cardiol.

[16] Mota FM, Araújo J, Arruda JA, Júnior HT, Pestana JO, de Sousa JM (2007). Clinical outcome of renal trans-plant patients after coronary stenting. Arq Bras Cardiol.

[17] Liu XF, Qin YW, Chen F, Zhang BL, Xu XD, Zhao XX (2013). Coronary angiography and percutaneous coronary intervention in kidney transplant patients, a report of 6 cases. Acad J Sec Mil Med Univ.

